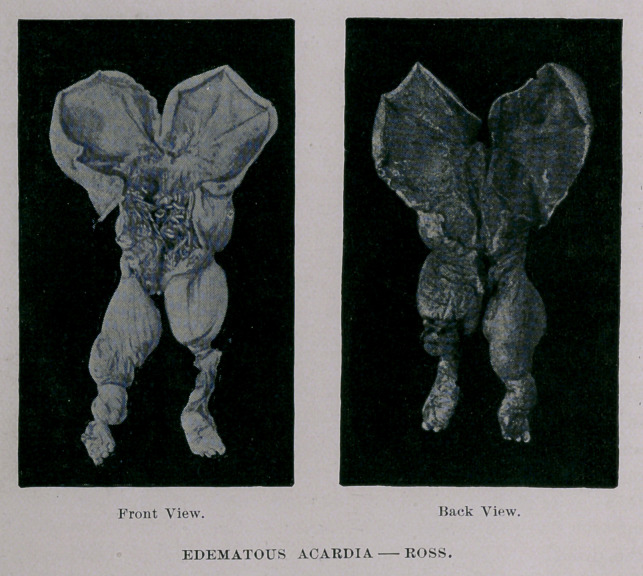# A Second Note on Croupous Rhinitis1Read before the Medical Society of the State of New York, February 4, 1891.

**Published:** 1891-04

**Authors:** Frank Hamilton Potter

**Affiliations:** Buffalo, N. Y., Lecturer on Diseases of the Nose and Throat in the Medical Department of Niagara University; Surgeon in charge of the Nose and Throat Department, Erie County Eye, Ear and Throat Infirmary; 273 Franklin Street


### A SECOND NOTE ON CROUPOUS RHINITIS.[1]

BY FRANK HAMILTON POTTER, M. D., Buffalo, N. Y.,

Lecturer on Diseases of the Nose and Throat in the Medical Department of Niagara University; Surgeon in charge of the Nose and Throat Department, Erie County Eye, Ear and Throat Infirmary.

Two years ago, before this Society, the writer read a paper with the title of Membranous Rhinitis.[2] At that time there was but little literature upon the subject, and he had not, therefore, the benefit of the experiences of others to enable him to test the conclusions there presented. Since then the disease has been described by many writers and has received a permanent place in our literature. It begins to appear in the text-books. Bosworth, for instance, in the last edition of his work on Diseases of the Nose and Throat, has a chapter with the title of Croupous Rhinitis." But by far the most exhaustive consideration of this

1. Read before the Medical Society of the State of New York, February 4, 1891.
2. Transactions of the Medical Society of the State of New York, 1889.

subject appears in a paper by Dr. V. Raulin,[1] of Bordeaux. From this paper we learn that the first notice of the disease we are considering appeared in 1882 in an article by Schüller.[2]

It thus appears, that as far as any record goes, the disease is of recent origin. It is probable, however, that it existed before Schüller described it, but without attracting attention. It deserves further study, and all cases observed should be recorded. The object in recurring to the subject at this time is to relate a single case which exhibited unusual complications, and to offer one or two suggestions which a larger observation has made pertinent.

In the first place, in regard to the name: Croupous Rhinitis seems from all points of view the better term, and moreover is the one employed by the majority of writers. The name used by the author in his first paper, while it indicates something of the nature of the disease, does not describe it as accurately as the word "Croupous." Next, as to the frequency of the disease: In the former article it is stated that this disease occurs in "about two per cent. of all cases of acute rhinitis." Increased experience convinces me that the proportion there stated is too great. Indeed, all those who have written on the subject and have noticed the author's contribution at all, have criticised this proportion of cases of croupous rhinitis to other cases of acute rhinitis as far out of the way. They are quite correct. Although that statement was in accordance with my experience at that time, the last two years convinces me that the statement that croupous rhinitis is a very rare disease would be nearer the truth. In this time I have seen but one case, which reduces the percentage very considerably. This case exhibited some unusual symptoms, and is, therefore, recorded in full. A man, 67 years of age, was referred to me by Dr. James W. Putnam, of Buffalo, on account of an acute rhinitis. He was a strong, healthy man, attended to business every day, and gave a personal history remarkably free from disease; the only thing bearing on his present attack being the statement that occasionally in the Fall of the year he had a "cold in the head," which was characterized by persistent watery discharge. He thought it was a kind of hay fever. It, however, did not appear regularly and it seemed to the writer that it was not sufficient to place him among the hay fever subjects. Upon examination he appeared to be suffering from an ordinary attack of acute rhinitis. I may state here that the nose was symmetrical and remarkably

1. *Revue de Laryngologie, D'Otologie, etc.*, May 1890, p. 289.
2. *Jahrbuch für Kinderheilkunde*, Jahr IV., p. 334.

Front View.

Back View.

EDEMATOUS ACARDIA — ROSS.

free from any deformity. There were no obstructions, either anteriorly or posteriorly. The nostrils, though, were not capacious, and from examination after the attack could be properly described as "narrow." Upon the second day afterward the croupous character of the disease became apparent. For a few days it ran the usual course and then appeared the complication unknown to me in connection with the disease. This consisted of edema first of the upper lip, then of the lower lip and upper part of the throat, and finally of the tongue. The latter swelled enormously and suddenly, and for a time we feared a serious result. Dr. Putnam, who lived directly opposite, was hurriedly called one night when this occurred and I believe his prompt action saved the case. Dreading that the edema would appear next in the epiglottis or in the laryngeal tissues, we prepared ourselves for that emergency, but fortunately this did not occur. Cathartics and diuretics were used freely and the patient was relieved, in this way, of large quantities of fluid. The amount of urine especially was very large, though I regret the daily quantity was not measured. The examination of the urine was negative so far as any disease of the kidneys was concerned. The patient was under observation twenty-five days and was then discharged fully recovered. We learn from this case that croupous rhinitis is not without its dangers, and that, therefore, every case ought to be carefully watched. Nothing has been said about the local treatment, because that was thoroughly considered in the former paper. The general plan is to relieve the stenosis and keep the nasal passages free from the membrane. The various measures that have been suggested may or may not be of benefit to a given case. The local treatment gives some relief, though it may be doubted whether, as yet, it has lessened the duration of the disease. It is to be hoped that continued observations will devise some method that will be more successful than any that, so far, has been suggested.

273 Franklin Street.

## Figures and Tables

**Figure f1:**